# Seasonal fluctuations attenuate stimulatory or inhibitory impacts of colonial birds on abundance, structure and diversity of soil biota

**DOI:** 10.3389/fmicb.2023.1080625

**Published:** 2023-05-03

**Authors:** Stanislav Pen-Mouratov, Tamar Dayan

**Affiliations:** School of Zoology and the Steinhardt Museum of Natural History, Tel Aviv University, Tel Aviv, Israel

**Keywords:** bird nesting area, bird roosting area, soil biota, soil nematode diversity, bird dropping, seasonal variation

## Abstract

Soil microorganisms and free-living nematodes were investigated in association with the nesting and roosting habitats of the following piscivorous and omnivorous colonial birds: black kite (*Milvus migrans*), great cormorant (*Phalacrocorax carbo*), black-crowned night heron (*Nycticorax nycticorax*) and little egret (*Egretta garzetta*), in Israel’s Mediterranean region. Abiotic variables, abundance, trophic structure, sex ratio and genus diversity of soil free-living nematodes, and total abundance of bacteria and fungi, were measured during the wet season, following our previous study conducted during the dry season. The observed soil properties were important drivers of soil biota structure. Presence of the most efficient elements for soil organisms, such as phosphorus and nitrogen, was strongly dependent on the diet of the compared piscivorous and omnivorous bird colonies; levels of these nutrients were notably higher in the bird habitats than in their respective control sites during the study period. Ecological indices showed that the different species of colonial birds can have different (stimulatory or inhibitory) impacts on abundance and diversity of the soil biota, affecting the structure of the soil free-living nematode population at the generic, trophic and sexual levels during the wet season. A comparison with results from the dry season illustrated that seasonal fluctuations can change, and even attenuate the effect of bird activity on the abundance, structure and diversity of the soil communities.

## 1. Introduction

Increasing allochtonous matter in birds’ nesting and roosting habitats has attracted the attention of researchers in the last decade (Anderson and Polis, 1999; Klimaszyk and Rzymski, 2013). Through the transport of minerals and nutrients in their guano, birds can be vital resource linkers, significantly affecting ecosystem functioning (Lundberg and Moberg, 2003; Fedriani et al., 2016). As an example, Murphy (1981) estimated that worldwide, seabirds transfer 10^4^–10^5^ tons of phosphorus (P) from sea to land annually *via* their guano. Bird feces have a noticeable impact on the environment, which can be felt thousands of kilometers away from the original source of the nutrients (Post et al., 1998; Ellis, 2005; Zmudczyńska et al., 2012). In addition, seasonal effects may significantly alter the impact of animal activity on soil properties and soil communities (Pen-Mouratov et al., 2019). Our previous investigation showed that soil properties and external environmental factors have a seasonal effect on the abundance and diversity of soil biota, as well as on microbial biomass and activity of soil microorganisms and their predators, the free-living nematodes (Pen-Mouratov et al., 2011). Moreover, soil property composition may be relatively uniformly distributed spatially and temporally throughout the wet season, with increasing and relatively high heterogeneity toward the dry season; this, in turn, reflects the structure and distribution of soil inhabitants (Pen-Mouratov et al., 2006). Information on the effects of colonial birds’ droppings on nematodes seems to be limited for many of the world’s regions, including the Mediterranean area of Israel; moreover, these effects have scarcely been examined from a seasonal point of view, leaving many open questions (Veresoglou et al., 2015). Approximately 500 million migratory birds annually find a temporary rest area in Israel (Yom-Tov et al., 2012; Krumenacker, 2013). This enormous number of birds has a significant effect on the soil biota (Pen-Mouratov and Dayan, 2019). Moreover, numerous studies have illustrated that soil microorganisms, along with soil free-living nematode communities, are among the best biological tools for assessing soil disturbances (e.g., Bongers, 1990; Pen-Mouratov et al., 2019) and play a key role in soil processes (Coleman and Hendrix, 2000).

The present study, conducted during the wet (WW) season, follows a previous one conducted during the dry (DH) season (Pen-Mouratov and Dayan, 2019). The same colonial bird species’ habitats were examined in both studies for their impact on soil microorganisms and soil free-living nematodes; only the season differed. The current study was aimed at determining seasonal fluctuations in the impact of excrement from different bird species. We hypothesized that seasonal fluctuations can change this impact (through changes in soil properties) on the abundance, structure and diversity of the soil microbial and free-living nematode communities. We further hypothesized that the wet season might even attenuate the impact of bird droppings on soil biota.

## 2. Materials and methods

### 2.1. Study site and location

The research area is located next to the Mediterranean Sea between the northern (32°33′25.834″N–34°55′32.476″E, Google Earth, 2015) and southern (31°26′23.897″N–34°27′30.622″E, Google Earth, 2015) points of the Mediterranean region of Israel. Israel’s area stretches 424 km from north to south, and its width ranges from 114 km (widest point) to 15 km (narrowest point). The northern and coastal regions of Israel are characterized by hot, dry summers and cool, rainy winters, whereas the southern and eastern areas are characterized by an arid climate. Average annual rainfall varies between <100 mm in the extreme south to about 1,100 mm in the north. Four different sites representing colonies of four different colonial birds with different dietary habits: (a) the black-crowned night heron, the little egret and the great cormorant (piscivorous – mainly fish eating) and (b) the black kite (omnivorous – broad, carnivorous diet Mebs and Schmidt, 2006; Laviad-Shitrit et al., 2017), were observed during the wet–warm (this study) and dry–hot (Pen-Mouratov and Dayan, 2019) periods of the year.

### 2.2. Sampling

The middle of each colony with an accumulation of observed birds (from 10 to 45 individuals per tree, on average) was chosen for soil sampling during the wet warm season of 2016, as the most favorable period for soil biota. Control sites, which had never been inhabited by the observed colonial birds, were selected close to the bird habitat sites. In each study area, four random 5 m × 5 m plots were chosen for soil sampling. Each soil sample, which consisted of five subsamples, was collected using a steel soil sampler (ø = 10 cm). Four random soil samples from the upper soil layer (depth of 0–5 cm) and four from the deeper soil layer (5–10 cm) were collected from the bird habitat (BH) sites and the corresponding control (CO) sites in the four sampling stations, giving 4 × 2 × 4 × 2 = 64 soil samples for the wet period. Added to the 64 samples from the dry period (Pen-Mouratov and Dayan, 2019), a total of 128 samples were compared.

### 2.3. Laboratory analysis

The collected soil samples were used for the following analyses: soil moisture was determined by oven-drying method (105°C, 48 h) to a constant weight; soil pH and electrical conductivity (EC, μS cm^−1^) were measured in soil extracts (1:2 soil-to-water ratio); soil chemicals were measured using the following standard methods: NO_3_, by ultraviolet spectrophotometry (Norman and Stucki, 1981); NH_4_, by calorimetric method (Grayer and Halmann, 1984); P by Murphy and Riley’s (1962) method; colony-forming units (CFU) of fungi and bacteria by plate count method (Pen-Mouratov et al., 2014). The soil free-living nematodes were extracted from 100-g soil samples using the Baermann funnel procedure (Cairns, 1960; van Bezooijen, 2006). The nematodes were counted, mounted in glycerin (Peraza-Padilla et al., 2017) and identified using a compound microscope (Bongers, 1994; Andrassy, 2009).

### 2.4. Ecological indices and statistical analysis

The seasonal impact of colonial birds on the abundance, structure, and diversity of soil nematode communities was assessed using the following parameters and ecological indices: (1) total number of nematodes per 100 g dry soil; (2) abundance of trophic nematode groups: bacteria-feeding, fungi-feeding, plant-parasitic and omnivore-predator; (3) trophic diversity, TD = (1/ΣPi^2^) (Heip et al., 1988); (4) Simpson’s dominance index, Dom = (ΣPi^2^) (Simpson, 1949); (5) Shannon–Weaver index, H′ = [−ΣPi (lnPi)] (Shannon and Weaver, 1949); (6) modified maturity index, MMI = (Σvi fi/n) (Bongers, 1990; Wasilewska, 1994; Yeates, 1994); (7) species richness, SR = [(S-1)/ln(N)] (Yeates and King, 1997); (8) basal index, BI = 100 × (b/b + e + s) (Ferris et al., 2001, 2004); (9) structure index, SI = 100 × [s/(s + b)] (Ferris et al., 2001, 2004); (10) enrichment index, EI = 100 × [e/(e + b)] (Ferris et al., 2001, 2004); (11) Wardle–Parkinson change index, V = [(2dBH/(dBH + dCO)) − 1], with BH = bird habitat and CO = control (Wardle and Parkinson, 1991).

All data were subjected to statistical analysis of variance (ANOVA) using the following SAS models: GLM, Duncan’s multiple-range test, nonparametric Kruskal–Wallis test and Pearson correlation coefficient were used to assess dissimilarity in soil variables between the bird habitat and the corresponding control areas during the observed seasons (SAS, 1988). Differences with *p* < 0.05 were considered significant. Multivariate redundancy analysis (RDA) was used to obtain additional data on the differences between the compared variables (CANOCO Program, Version 4.54, October 2005 – written by ter Braak (©)1988–2005; ter Braak and Prentice, 1996).

## 3. Results

### 3.1. Soil properties

During the observed wet period, no significant differences (*p* > 0.05) were found in soil moisture content between the inhabited sites and their respective control sites, except for the little egret habitat (both soil layers) and black-crowned night heron habitat (deeper soil layer; Table 1). Soil pH showed lower values in all of the observed inhabited areas, except for the black kite habitat, where pH was significantly higher compared to its control site (Table 1). Soil EC was higher in all bird-inhabited vs. control sites in the two soil layers, except for black-crowned night heron (deeper soil layer; Table 1). Soil NO_3_ and NH_4_ contents were lower at all control sites vs. their respective bird-inhabited sites (except NH_4_ for the deeper soil layer of the black kite site; Table 1).

**Table 1:** Changes in soil properties and abundance of soil biota in colonial birds’ habitat and control sites during the wet period.

Bird species	Location	SM	pH	EC	NO_3_	NH_4_	P	Bacteria	Fungi	Nematodes
				Upper soil layer						
Little egret	Bird habitat	7.57 ± 1.7***	7.45 ± 0.04***	2.12 ± 0.37***	4.46 ± 0.6***	5.06 ± 0.3***	65.63 ± 2.9***	24.87 ± 5.4*	9.10 ± 3.7	1124.63 ± 796.3^KW*^
Little egret	Control site	28.02 ± 3.8	7.94 ± 0.05	0.50 ± 0.06	0.71 ± 0.3	0.18 ± 0.05	17.43 ± 6.5	14.61 ± 5.2	5.08 ± 0.6	232.14 ± 111.9
Black-crowned night heron	Bird habitat	16.12 ± 2.0	7.65 ± 0.03***	1.28 ± 0.24**	9.27. ± 0.7***	1.29 ± 0.08***	40.42 ± 0.99***	21.75 ± 3.3	13.13 ± 6.3	611.68 ± 684.9
Black-crowned night heron	Control site	24.45 ± 8.5	7.95 ± 0.06	0.62 ± 0.18	0.89 ± 0.02	0.07 ± 0.02	17.89 ± 4.4	18.84 ± 11.2	6.34 ± 2.3	367.97 ± 263.4
Great cormorant	Bird habitat	29.86 ± 6.9	6.20 ± 0.36***	3.82 ± 0.23***	11.55 ± 1.07**	2.93 ± 0.4***	59.21 ± 6.8**	9.18 ± 1.6**	0.98 ± 0.2***	2019.79 ± 1487.3
Great cormorant	Control site	21.07 ± 8.2	7.97 ± 0.12	0.57 ± 0.08	5.62 ± 0.6	0.4 ± 0.26	31.22 ± 5.5	24.44 ± 4.2	6.09 ± 1.5	745.95 ± 690.7
Black kite	Bird habitat	11.35 ± 6.4	8.36 ± 0.31 ^*^	0.47 ± 0.09*	2.74 ± 0.08***	0.63 ± 0.06***	32.20 ± 2.19***	10.46 ± 5.33*	0.94 ± 0.2**	223.6 ± 96.1
Black kite	Control site	6.81 ± 3.9	7.97 ± 0.02	0.33 ± 0.04	0.06 ± 0.01	0.06 ± 0.02	12.71 ± 0.74	2.72 ± 0.91	0.34 ± 0.1	674.5 ± 690.1
				Deeper soil layer						
Little egret	Bird habitat	9.83 ± 3.0*	7.71 ± 0.06**	0.82 ± 0.13**	1.12 ± 0.3**	0.52 ± 0.5^KW*^	36.12 ± 3.4***	9.58 ± 2.1	2.64 ± 1.1	340.21 ± 82.6
Little egret	Control site	26.67 ± 9.8	7.91 ± 0.03	0.41 ± 0.08	0.21 ± 0.07	0.02 ± 0.004	6.80 ± 4.1	8.28 ± 1.8	2.28 ± 0.5	579.76 ± 369.4
Black-crowned night heron	Bird habitat	18.81 ± 4.8**	7.94 ± 0.09*	0.88 ± 0.32	1.67 ± 0.46**	1.13 ± 0.02***	19.61 ± 0.8**	15.01 ± 3.5	11.50 ± 4.9*	387.87 ± 426.9
Black-crowned night heron	Control site	31.06 ± 1.8	8.10 ± 0.04	0.63 ± 0.04	1.44 ± 0.08	0.004 ± 0.0002	9.76 ± 4.3	14.90 ± 7.4	4.88 ± 1.3	244.44 ± 61.1
Great cormorant	Bird habitat	31.57 ± 10.9	7.10 ± 0.17***	1.77 ± 0.18***	8.10 ± 0.64***	0.29 ± 0.03***	39.37 ± 8.2**	2.35 ± 2.0**	0.68 ± 0.2^KW*^	866.66 ± 395.8^KW*^
Great cormorant	Control site	25.90 ± 10.4	8.00 ± 0.10	0.48 ± 0.09	1.44 ± 0.05	0.03 ± 0.02	12.21 ± 2.3	8.90 ± 1.8	2.66 ± 1.8	473.38 ± 94.3
Black kite	Bird habitat	8.80 ± 3.3	8.38 ± 0.20**	0.40 ± 0.05**	0.66 ± 0.2***	0.036 ± 0.01	16.09 ± 4.2*	16.12 ± 5.9**	0.32 ± 0.1*	305.81 ± 87.4
Black kite	Control site	9.21 ± 3.3	7.93 ± 0.06	0.28 ± 0.02	0.02 ± 0.01	0.02 ± 0.02	8.50 ± 1.8	1.70 ± 0.8	0.11 ± 0.01	431.17 ± 305.8

Soil properties: SM, soil moisture; pH, soil acidity and alkalinity; EC, electrical conductivity (dS/m); NO_3_, nitrate (mg/100 g dry soil); NH_4_, ammonium cation (mg/100 g dry soil); P, phosphorus (mg/100 g dry soil). Soil biota: bacteria, colony-forming units (CFUs) of bacteria (*B*, *n* × 10^6^ CFU per g dry soil) and fungi (*F*, *n* × 10^6^ CFU per g dry soil); nematodes, total mnumber of nematode individuals (per 100 g dry soil). One-way ANOVA, and ^KW^, nonparametric Kruskal-Wallis test (*p < 0.05; **p < 0.01; ***p < 0.001, *n* = 64) are indicated between all compared differences Boxed values indicate higher values of soil properties and soil biota in the control sites compared to the their corresponding bird habitat.

### 3.2. Colony-forming units of fungi and bacteria

During the wet period, the differences in bacterial CFU in the upper soil layer between all bird-inhabited and control sites (except for black-crowned night heron) were significant (Table 1). However, in the deeper soil layer, bacterial CFU differed between bird-inhabited and control sites only for the great cormorant and black kite (Table 1). Fungal CFU differed between bird-inhabited and control sites for the great cormorant and black kite in both soil layers, and for the black-crowned night heron in the deeper soil layer (Table 1). Fungal CFU were higher in the control vs. bird-inhabited sites for the great cormorant in both soil layers, whereas at the other observed sites, fungal CFU were either higher or not different in the bird-inhabited vs. respective control site (Table 1).

Bacterial CFU showed no correlation with soil moisture content during the observed wet period, whereas fungal CFU was negatively correlated with soil moisture values in the great cormorant and black-crowned night heron control areas (Table 2). Bacterial and fungal CFU were negatively correlated with pH values in the following areas: for bacteria – little egret habitat and great cormorant habitat, and for fungi – great cormorant habitat. The number of bacteria was positively correlated with EC in the little egret and great cormorant habitat areas (Table 2). The number of fungi was correlated with EC in the black-crowned night heron control area (Table 2).

**Table 2:** Pearson correlation coefficient between soil properties and soil biota in the birds’ habitats and control sites during the wet period.

Bird species	Location	Bacteria	Fungi	Nematodes	Male (nematodes)	Female (nematodes)	Juvenile (nematodes)
Little egret	Bird habitat	pH^−0.84**^, EC^0.73*^, NH_4_^0.94**^	NH_4_^0.82*^, P^0.77*^, B^0.92**^	EC^0.74*^			
-"-	-"-	NO_3_^0.83*,^ P^0.88**^, F^0.92**^					
Little egret	Control site	F^0.83*^	NH_4_^0.83*^, B^0.83^		SM^−0.73*^		
Black-crowned night heron	Bird habitat	NH_4_^0.75*^, P^0.74*^		F^0.75*^		F^0.87**^	
Black-crowned night heron	Control site	P^0.71*^	EC^−0.84**^, SM^−0.82*^			EC^0.73*^	
Great cormorant	Bird habitat	pH^−0.80*^, EC^0.86**,^ NH_4_^0.89**^, NO_3_^0.79^	pH^−0.71*^,P^0.82*^, NO_3_^0.77^				
Great cormorant	Control site	NO_3_^0.91**^, P^0.83*^, F^0.90**^	NO_3_^0.73*^, B^0.90**^, SM^−0.75*^	SM^−0.73*^	F^0.76*^, SM^−0.84**^, pH^−0.81*^	SM^−0.86**^	
Black kite	Bird habitat		NH4^0.91**^,NO_3_^0.76*^, P^0.86**^				
Black kite	Control site	NO_3_^0.92**^		NO_3_^0.77*,^ B^0.90**^	EC^0.86**^	NO_3_^0.72*^, B^0.91**^	NO_3_^0.76*^, B^0.85**^

Soil properties: SM, soil moisture; pH, soil acidity and alkalinity; EC, electrical conductivity (dS/m); NO_3_, nitrate (mg/100 g dry soil); NH_4_, ammonium cation (mg/100 g dry soil); P, phosphorus (mg/100 g dry soil). Soil biota: bacteria – colony-forming units (CFUs) of bacteria (*B*, *n* × 10^6^ CFU per g dry soil) and fungi (*F*, *n* × 10^6^ CFU per g dry soil); nematodes – total mnumber of nematode individuals (per 100 g dry soil). *, ** Correlation coefficients significant at *p* < 0.05 and 0.01, respectively (*n* = 64).

Bacterial and fungal CFU demonstrated the following positive correlations with NH_4_ during the wet period: for bacteria – little egret habitat, great cormorant habitat and black-crowned night heron habitat; for fungi – little egret habitat, little egret control and black kite habitat. No correlation between bacterial CFU and NH_4_ was found at the control sites (Table 2). Bacterial and fungal CFU were positively correlated with NO_3_ in the following areas: for bacteria – little egret habitat, great cormorant habitat, great cormorant control site, and black kite control site; for fungi – great cormorant habitat, great cormorant control site and black kite habitat (Table 2). The observed microorganisms showed a positive correlation with P in the following bird-inhabited areas: bacteria – little egret and black-crowned night heron (Table 2); fungi – little egret, great cormorant and black kite (Table 2). In addition, the number of bacteria was positively correlated with P in the great cormorant and black-crowned night heron control sites (Table 2).

The Wardle–Parkinson change index revealed both stimulatory and inhibitory effects of the observed birds on soil microorganisms in the study area during the wet period (Table 3). Among the observed colonial birds, great cormorant activity had the strongest inhibitory effect on soil microorganisms (Table 3). Little egret exerted a stimulatory or neutral effect (Table 3). Black-crowned night heron and black kite colonies had mainly positive effects on soil microorganisms (Table 3).

**Table 3:** Wardle-Parkinson change index (*V*) indicates effects of nesting birds’ droppings on abundance of soil biota during the wet period.

Soil biota	Depth	Bird 1	Bird 2	Bird 3	Bird 4
Bacteria	Upper soil layer	SS (0.27)	MI (−0.45)	SS (0.14)	MS (0.54)
-"-	Deeper soil layer	NC (0.07)	MI (−0.62)	NC (0.05)	ES (0.80)
Fungi	Upper soil layer	SS (0.25)	EI (−0.71)	SS (0.31)	MS (0.47)
-"-	Deeper soil layer	NC (0.05)	MI (−0.50)	MS (0.36)	MS (0.45)
Nematodes	Upper soil layer	ES (0.66)	MS (0.46)	SS (0.25)	MI (−0.50)
-"-	Deeper soil layer	SI (−0.26)	SS (0.29)	SS (0.23)	SI (−0.17)

All negative values in this table are marked with a dark background. Soil biota: bacteria – colony-forming units (CFUs) of bacteria (*B*, *n* × 10^6^ CFU per g dry soil) and fungi (*F*, *n* × 10^6^ CFU per g dry soil); nematodes – total mnumber of nematode individuals (per 100 g dry soil). Bird species: bird 1, little egret (*Egretta garzetta*); bird2, black-crowned night heron (*Nycticorax nycticorax*); bird 3, great cormorant (Phalacrocorax carbo); bird 4, black kite (*Milvus migrans*). Warde-Parkinson change index values: EI, extreme inhibition (*V* < −0.67); MI, moderate inhibition (−0.33 > *V* > −0.67); SI, slight inhibition (−0.05 > *V* > −0.33); NC, no change; (−0.05 < *V* < 0.05); SS, slight stimulation (0.05 < *V* < 0.33); MS. Moderate stimulation (0.33 < *V* < 0.67); ES, extreme stimulation (*V* > 0.67).

### 3.3. Nematode community structure

During the wet period, total number of nematodes was higher in the little egret (upper soil layer) and great cormorant (deeper soil layer) habitats, but did not differ for the other observed sites (Table 1). Total number of nematodes was negatively correlated with soil moisture (great cormorant control site) and positively correlated with bacterial CFU (black kite control site), fungal CFU (black-crowned night heron habitat) and NO_3_ (black kite control site; Table 2). As with the soil microorganisms, the Wardle–Parkinson change index revealed both stimulatory and inhibitory effects of the observed birds on total number of nematodes (Table 3). Only black kite exerted an inhibitory effect on the total number of nematodes in the deeper soil layer; the great cormorant and black-crowned night heron had a stimulatory effect in both soil layers, and little egret had a stimulatory effect in the upper soil layer (Table 3).

**Figure 1:**
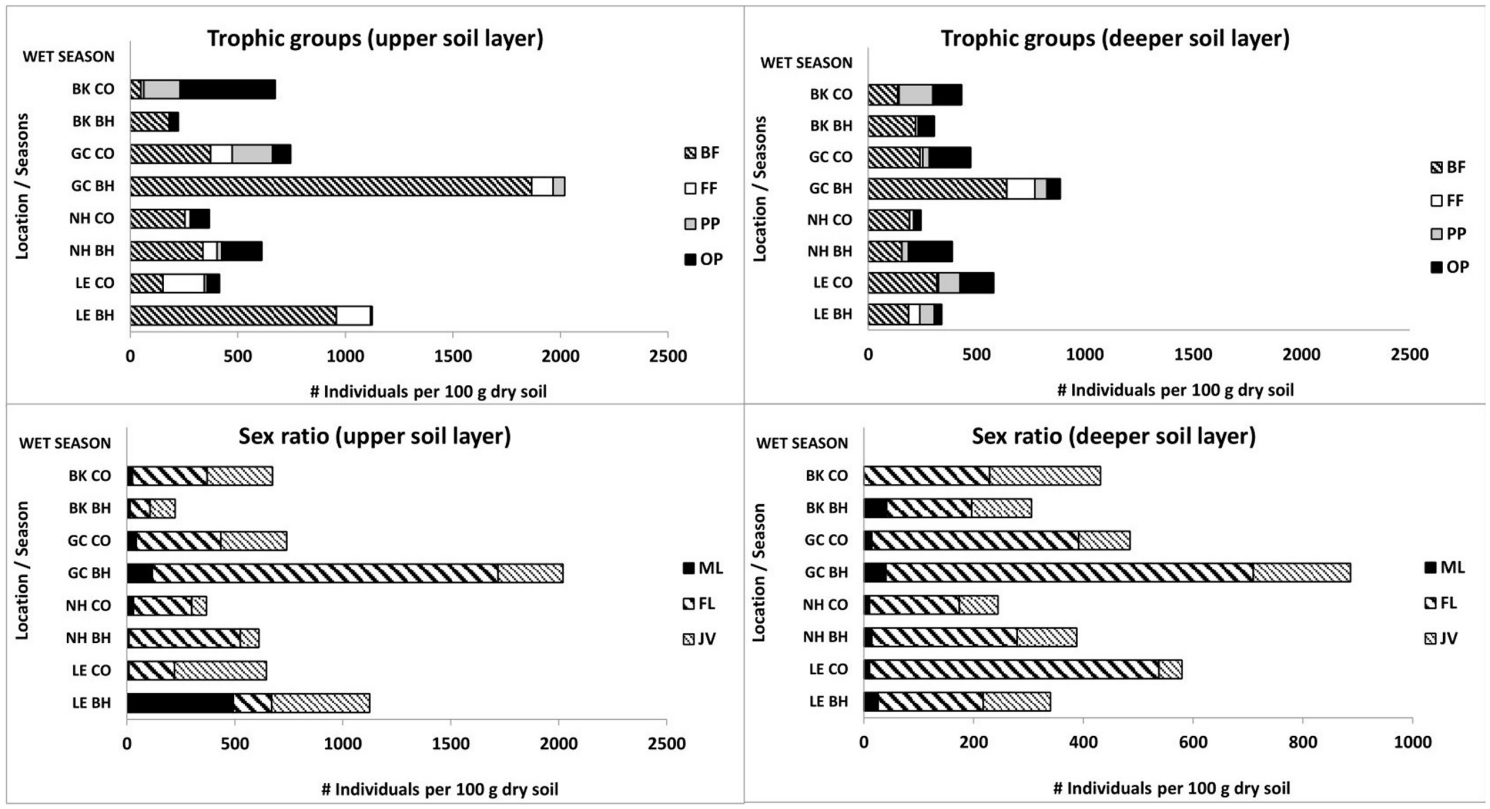
Changes in trophic groups and sex ratio of soil free-living nematodes in colonial birds’ habitat (BH) and control (CO) sites during the wet period. Bird species: LE, little egret (*Egretta garzetta*); NH, black-crowned night heron (*Nycticorax nycticorax*); GC, great cormorant (*Phalacrocorax carbo*); BK, black kite (*Milvus migrans*). Trophic groups of nematodes: BF, bacteria-feeding; FF, fungi-feeding; PP, plant-parasitic; OP, omnivore-predator. Sex groups of nematodes: ML, male; FL, female; JV, juvenile.

We identified 61 nematode taxa in the present study: 21 belonged to the bacteria-feeding trophic group, 7 were fungi-feeding nematodes, 15 were plant-parasitic nematodes, and 18 were omnivore-predator nematodes (Table 4). Moreover, *Panagrolaymus* and *Mesorhabtidae* dominated during the wet period (Table 4). Along with the total number of nematodes, bird activity was found to affect the trophic and sexual structure of the nematode community (Figure 1).

**Table 4:** Relative dominance of soil nematodes and correlation coefficient between soil free-living nematode genera and the observed soil properties in the upper and deeper soil layers in the colonial birds’ habitats (and control sites during the wet study period).

	^a^c-p values	Wet period		Bird habitat						Control area					
		Habitat	Conrol	SM	pH	EC	NO_3_	NH_4_	P	SM	pH	EC	NO_3_	NH_4_	P
Bacterivores															
*Achromadora*	3	+													
*Acrobeles*	2	+++	++	W^0.57**^											
*Acrobeloides*	2	+++	++	W^−0.47**^								W^0.36*^			
*Cephalobus*	2	+++	++					W^0.40*^				W^0.47**^			
*Cervidellus*	2	+	+					W^0.50**^	W^0.40*^			W^0.52**^			
*Chiloplacus*	2	++	+									W^−0.46**^			
*Chromadorinae*	3	+	+												
*Chronogaster*	3		+								W^0.53**^	W^0.35*^			
*Eucephalobus*	2	+	+												
*Eumonhystera*	2	+	+			W^0.36*^					W^−0.38*^		W^0.46**^		
*Heterocephalobus*	2	+													
*Metoteratocephalus*	3		+									W^0.36*^			
*Mesorhabtidae*	1	++++	+	W^0.55**^	W^−0.62**^	W^0.66**^	W^0.49**^				W^−0.36*^		W^0.43*^		
*Monhystera*	2	+	++										W^0.76**^	W^0.77**^	W^0.57**^
*Panagrolaimus*	1	+++++	+		W^−0.41*^	W^0.60**^	W^0.40*^	W^0.46**^	W^0.48*^						
*Plectus*	2	+	+												
*Prismatolaimus*	3	+	+++							W^0.36*^					
*Rhabditis*	1		+										W^0.46**^		
*Teratocephalus*	3		+												
*Tylocephalus*	2	+	+												
*Wilsonema*	2	+	+												
Fungivores															
*Aphelenchoides*	2	++	+					W^0.48**^	W^0.38*^						
*Aprutides*	2		+												
*Aphelenchus*	2	++	+				W^0.37*^						W^0.41*^	W^0.42*^	
*Ditylenchus*	2	+	+		W^−0.36*^								W^0.46**^		
*Nothotylenchus*	2	+			W^−0.44*^	W^0.39*^									
*Paraphelenchus*	2	+													
*Tylencholaimus*	4		+												
Plant-parasites															
*Criconema*	3		+												
*Dolichorhynchus*	3		+									W^−0.38*^			
*Filenchus*	2	+	++										W^0.48**^		
*Helicotylenchus*	3		+												
*Longidorella*	4	+	+	W^0.52**^	W^−0.38*^		W^0.36*^						W^0.43*^		
*Longidorus*	5	+	+							W^0.39*^					
*Malenchus*	2		+												
*Meloidogyna*	3		+									W^−0.40*^			
*Pratylenchoides*	3		+												
*Pratylenchus*	3		+												
*Rotylenchus*	3		+												W^−0.36*^
*Trichodorus*	4		+												
*Trophurus*	3		+												
*Tylenchorhynchus*	3	+	++				W^−0.37*^			W^−0.36*^		W^−0.42*^			
*Tylenchus*	2		+									W^−0.35*^			
Omnivores-predators															
*Aporcelaimellus*	5	+	+++									W^−0.38*^			
*Aporcelaimus*	5	+	+												
*Aporcelaimoides*	5	+	++												
*Aporcelaimoides*	5		+									W^−0.43*^			
*Clarcus*	4		+												
*Discolaimium*	5	+	+												
*Discolaimoides*	5	+	+							W^−0.37*^		W^−0.37*^			
*Discolaimus*	5		+												
*Dorylaimellus*	5		+												
*Dorylaimoides*	4	++	+												
*Dorylaimus*	4		+												
*Mesodorylaimus*	5	+	+												
*Microdorylaimus*	4	++	++												
*Milonchulus*	4		+												
*Nygolaimus*	5	+	+							W^−0.35*^					
*Pungentus*	4		+												
*Thonus*	4	+	+												
*Tripyla*	3		+												

All negative values in this table are marked with a dark background. +++++, eudominant (>10%); ++++, dominant (5–10%); +++, subdominant (2–5%); ++, resident (1–2%); +, subresident (<1%). Soil properties: SM, soil moisture; pH, soil acidity and alkalinity; EC, electrical conductivity (dS/m); NO_3,_ nitrate (mg/100 g dry soil); NH_4_, ammonium cation (mg/100 g dry soil); P, phosphorus (mg/100 g dry soil). *, ** Correlation coefficients significant at *p* < 0.05 and 0.01, respectively (*n* = 64).

a c–p values, characterized by life history characteristics, are adapted from Bongers (1990).

The data obtained during the wet period showed significantly higher mean amounts of bacteria-feeding nematodes in the upper soil layer of the little egret habitat (one-way ANOVA, *p* < 0.05) than in the other observed sites with no differences between bird and control sites in the deeper soil layer (Figure 1). The total number of fungi-feeding nematodes was significantly higher (one-way ANOVA, *p* < 0.05) in the deeper soil layer of the great cormorant habitat vs. control sites (Figure 1). The total number of omnivore-predator nematodes only showed differences between comparable sites (one-way ANOVA) in the upper soil layer for little egret control site vs. habitat (*p* < 0.05; Figure 1). However, Kruskal–Wallis nonparametric test (*p* < 0.05) revealed effects of bird activity on the plant-parasitic and omnivore-predator nematodes as well (black kite habitat and great cormorant habitat, upper soil layers; Figure 1). In addition, bacteria-feeding nematodes showed a negative correlation with soil moisture (great cormorant control site) and a positive correlation with EC (little egret habitat and black-crowned night heron control site), NO_3_ (little egret habitat) and fungal CFU (black-crowned night heron habitat), whereas fungi-feeding nematodes only showed a positive correlation with NH_4_ in the control sites; plant-parasitic nematodes showed a negative and positive correlation with soil moisture (black-crowned night heron control site and black kite control site, respectively) and a positive correlation with bacterial and fungal CFU (black kite control site and black-crowned night heron control site, respectively), whereas omnivore-predator nematodes showed a positive correlation only with bacterial CFU and NO_3_ in the black kite control site and a negative correlation with NH_4_, P (little egret habitat), NO_3_ (little egret habitat and control site, great cormorant control site and black kite control site), bacterial CFU (great cormorant control site and black kite control site) and fungal CFU (great cormorant habitat).

Twenty-five and thirty-three nematode species, mainly from the bacteria-feeding and fungi-feeding trophic groups, were correlated with soil properties in the bird habitats and control sites, respectively (Table 4). In other words, there were notably less correlation events between soil nematodes and soil properties in the bird habitats vs. control sites. Furthermore, there were differences in the negative correlation events between soil nematodes and the observed chemicals at the study sites, with a higher number of negative correlation events in the control sites vs. bird habitats (Table 4). In addition, *Acrobeloides* showed a negative correlation with soil moisture, whereas *Acrobeles*, *Mesorhabtidae* and *Longidorella* were positively correlated with soil moisture in the bird habitats; *Tylenchorhynchus*, *Discolaimoides* and *Nygolaimus* showed a negative correlation with soil moisture, whereas *Prismatolaimus* and *Longydorus* showed a positive correlation with soil moisture in the control sites (Table 4). *Mesorhabtidae*, *Panagrolaimus*, *Ditylenchus*, *Nothotylenchus* and *Longidorella* in the bird habitat areas, and *Eumonhystera* and *Mesorhabtidae* in the control sites showed a negative correlation with pH (Table 4). *Eumonhystera*, *Mesorhabtidae*, *Panagrolaimus* and *Nothotylenchus* in the bird habitats, and *Acrobeloides*, *Cephalobus*, *Cervidellus*, *Chronogaster* and *Metoteratocephalus* showed a positive correlation with EC in the control sites (Table 4); *Chiloplacus*, *Dolichorhynchus*, *Meloidogyna*, *Tylenchorhynchus*, *Tylenchus*, *Aporcelaimellus*, *Aporcelaimoides* and *Discolaimoides* were negatively correlated with EC in the control sites (Table 4). Negative correlations were found for *Tylenchorhynchus* with NO_3_ in the bird habitats and *Rotylenchus* with P in the control sites (Table 4). All other correlation events between the nutrients and the observed soil nematodes were positive (Table 4).

During the wet period, the number of nematode males was negatively correlated to soil moisture (little egret control and great cormorant control sites) and pH (great cormorant control site), and positively correlated to EC (black kite control site) and fungal CFU (great cormorant control site; Table 2). The number of nematode females was negatively correlated to soil moisture (great cormorant control site), and positively correlated to EC (black-crowned night heron control site), bacterial CFU (black kite control site) and fungal CFU (black-crowned night heron habitat; Table 2). The total number of juveniles showed a positive correlation only with bacterial CFU (black kite control site; Table 2). The observed elements NO_3_, NH_4_ and P did not exert any significant effect on the nematode sex ratio, and only females and juveniles showed a correlation with NO_3_ (black kite control site) during the wet period (Table 2).

### 3.4. Ecological indices

During the wet period, trophic diversity index values were significantly higher in the upper soil layer of the control site vs. bird habitat for little egret, great cormorant and black kite, with no difference in the deeper soil layer (Table 5). Trophic diversity index showed positive (black kite-inhabited site) or negative (little egret control site) correlations with soil moisture, a negative correlation with EC (little egret habitat), a positive correlation with fungal CFU at the little egret-inhabited site, and a negative correlation with fungal CFU (great cormorant habitat), bacterial CFU (little egret habitat) and the observed nutrients (little egret habitat; Table 6). Simpson’s dominance index values were significantly higher only for black kite-inhabited vs. black kite control sites (Table 5). They showed a negative correlation with pH (little egret control site; Table 6). Shannon–Weaver index values were significantly higher only in the upper soil layer of the control vs. bird sites for little egret and black kite (Table 5). They only showed a positive correlation to soil moisture (black kite-inhabited and black kite control sites) and were negatively correlated with bacterial CFU, NH_4_ and P for the little egret habitat (Table 6). The modified maturity index was higher for little egret and great cormorant control sites in both soil layers (Table 5). This index was positively correlated with pH (little egret habitat), negatively correlated with EC (little egret habitat and great cormorant habitat), bacterial CFU (little egret habitat and great cormorant control site), NH_4_ (little egret habitat, little egret control site and great cormorant habitat), NO_3_ (little egret habitat, little egret control site, great cormorant habitat and great cormorant control site) and P (little egret habitat, great cormorant habitat and control site), and negatively correlated with fungal CFU (little egret habitat, great cormorant habitat and great cormorant control site; Table 6). Species richness values were only higher in the upper soil layer for little egret and black kite control vs. inhabited sites (Table 5), and showed a positive correlation with pH (little egret habitat) and a negative correlation with EC, bacterial CFU and all observed nutrients in the little egret habitat (Table 6). Basal index was lower in the upper soil layer of the bird-inhabited vs. control site for great cormorant, and about five times higher in the deeper soil layer of the bird-inhabited vs. control site for little egret (Table 5). Basal index values were negatively correlated with soil moisture (black kite-inhabited sites), EC (great cormorant habitat), fungal CFU and NH_4_ (little egret and great cormorant habitats), negatively or positively correlated with pH (little egret habitat and black kite control site, respectively) and P (black-crowned night heron and great cormorant control sites, respectively), and positively correlated with NO_3_ (great cormorant control site; Table 6). Structure index values were significantly higher in both soil layers of the control vs. bird-inhabited sites for little egret and great cormorant (Table 5). These values were negatively or positively correlated with soil moisture (black-crowned night heron habitat and black kite habitat, respectively) and negatively correlated with bacterial CFU (great cormorant control site), fungal CFU (great cormorant habitat) and NO_3_ content (little egret control site; Table 6). The enrichment index showed higher values for both soil layers of the black-crowned night heron and great cormorant habitat vs. control sites, and lower values in the deeper soil layer for the black kite habitat vs. control site (Table 5). This index showed a negative correlation with pH (little egret habitat and great cormorant habitat), and positive correlations with bacterial CFU (little egret habitat, and great cormorant habitat and control site) and fungal CFU (little egret habitat and control site, black kite habitat) and with NH_4_ (little egret habitat, great cormorant habitat and black kite habitat), NO_3_ (little egret habitat, great cormorant habitat and black kite habitat) and P (little egret and black kite habitats; Table 6).

**Table 5:** Changes in values of the ecological indices in the study area during the wet period.

Bird species	Location	TD	Dom	H′	MMI	SR	BI	SI	EI
					Upper soil layer				
Little egret	Bird habitat	1.23*	0.45	1.06 *	1.44**	0.43*	16.6	17.3*	83.4
Little egret	Control site	2.1	0.19	1.84	2.84	1.71	9.03	87.5	78.2
Black-crowned night heron	Bird habitat	2.01	0.21	1.74	2.96	1.11	16.6	88.4	83.0^*^
Black-crowned night heron	Control site	2.23	0.15	3.5	2.59	1.13	21.2	40.8	27.6
Great cormorant	Bird habitat	1.27*	0.41	1.08	1.30*	0.47	22.7*	0 *	93.1*
Great cormorant	Control site	2.37	0.28	1.42	2.39	1.92	34.1	45.6	31.4
Black kite	Bird habitat	1.49*	0.33^KW*^	1.22*	2.22	0.57 **	34.47	39.6	51.5
Black kite	Control site	2.04	0.15	2.02	3.42	1.31	13.08	72.2	41.6
					Deeper soil layer				
Little egret	Bird habitat	2.31	0.18	1.92	2.38**	1.46	36.69**	46.0 *	32.5
Little egret	Control site	2.44	0.25	1.85	3.21	1.58	7.22	92.7	25
Black-crowned night heron	Bird habitat	1.71	0.22	2.33	2.97	1.01	8.83	86	82.1*
Black-crowned night heron	Control site	1.51	0.29	1.56	2.39	1.01	45.7	48.4	32.4
Great cormorant	Bird habitat	1.89	0.32	1.27	2.24*	0.79	47.4	20.4*	44.0**
Great cormorant	Control site	2.34	0.16	1.73	3.01	1.22	18.9	80.8	3.9
Black kite	Bird habitat	1.74	0.29	1.19	2.83	0.61	41.5	58.4	0^*KW^
Black kite	Control site	2.29	0.16	2.03	3.09	1.59	21.1	77.6	27.9

Ecological indices: TD, trophic diversity; Dom, Simpson’s dominance index; H′, Shannon-Weaver index; MMI, modified maturity index; SR, species richness; BI, basal index; SI, structure index; EI, enrichment index. One-way ANOVA, and nonparametric Kruskal–Wallis test (*p < 0.05; **p < 0.01; ***p < 0.001, *n* = 128) were used to indicate differences between the compared bird habitat and control sites.

**Table 6:** Pearson correlation coefficient between ecological indices, soil properties and soil biota in the birds’ habitat and control sites during the wet period.

Bird species	Location	TD	Dom	H′	MMI	SR	BI	SI	EI
Little egret	Bird habitat	B^−0.85**^, F^0.71*^, EC^−0.82*^, pH^0.76*^,	B^0.80*^	B^−0.84**^, NH_4_^−0.75*^, P^−0.78*^	B^−0.89**^, F^−0.75*^, EC^−0.80*^, pH^0.76*^,	B^−0.82*^, EC^−0.83*^, pH^0.78*^,	B^−0.86**^, NH_4_^−0.77*^,pH^0.76*^		B^0.89**^, F^0.74*^, EC^0.76*^, pH^−0.81*^,
-"-	-"-	NH_4_^−0.87**^, NO_3_^−0.84**^, P^−0.94**^			NH_4_^−0.89**^, NO_3_^−0.85**^, P^−0.93**^	NH_4_^−0.87**^, NO_3_^−0.85**^, P^−0.93**^			NH_4_^0.89**^, NO_3_^0.84**^, P^0.90**^
Little egret	Control site	SM^−0.74*^	SM^0.80*^, pH^−0.72*^		NH_4_^−0.75*^, NO_3_^−0.90**^			NO_3_^−0.71*^	F^0.88**^, EC^0.79*^
Black-crowned night heron	Bird habitat							SM^−0.71*^	
Black-crowned night heron	Control site						P^−0.72*^		EC^0.71*^
Great cormorant	Bird habitat	F^−0.72*^,			F^−0.75*^, EC^−0.81*^, NH_4_^−0.76*^, NO_3_^−0.76*^, P^−0.79*^		B^−0.84**^, EC^−075*^, NH_4_^−0.76*^	F^−0.72*^	B^0.85**^, EC^0.90**^, pH^−0.74*^,NH_4_^0.91**,^ NO_3_^0.78^
Great cormorant	Control site				B^−0.86**^, F^−0.71*^, NO_3_^−0.79*^, P^−073*^		NO_3_^0.74*^, P^0.74*^	B^−0.80*^	B^0.78*^
Black kite	Bird habitat	SM^0.74*^		SM^0.74*^			SM^−0.85**^	SM^0.84**^	F^0.86**^, NH_4_^0.98**,^ NO_3_^0.89**^, P^0.94**^
Black kite	Control site			SM^0.73*^			pH^−0.75*^		

Soil properties: SM, soil moisture; pH, soil acidity and alkalinity; EC, electrical conductivity (dS/m); NO_3_, nitrate (mg/100 g dry soil); NH_4_, ammonium cation (mg/100 g dry soil); P, phosphorus (mg/100 g dry soil). Ecological indices: TD, trophic diversity; Dom, Simpson’s dominance index; H′, Shannon-Weaver index; MMI, modified maturity index; SR, species richness; BI, basal index; SI, structure index; EI, enrichment index. *, ** Correlation coefficients significant at *p* < 0.05, 0.01, and 0.0001, respectively (*n* = 64).

**Figure 2:**
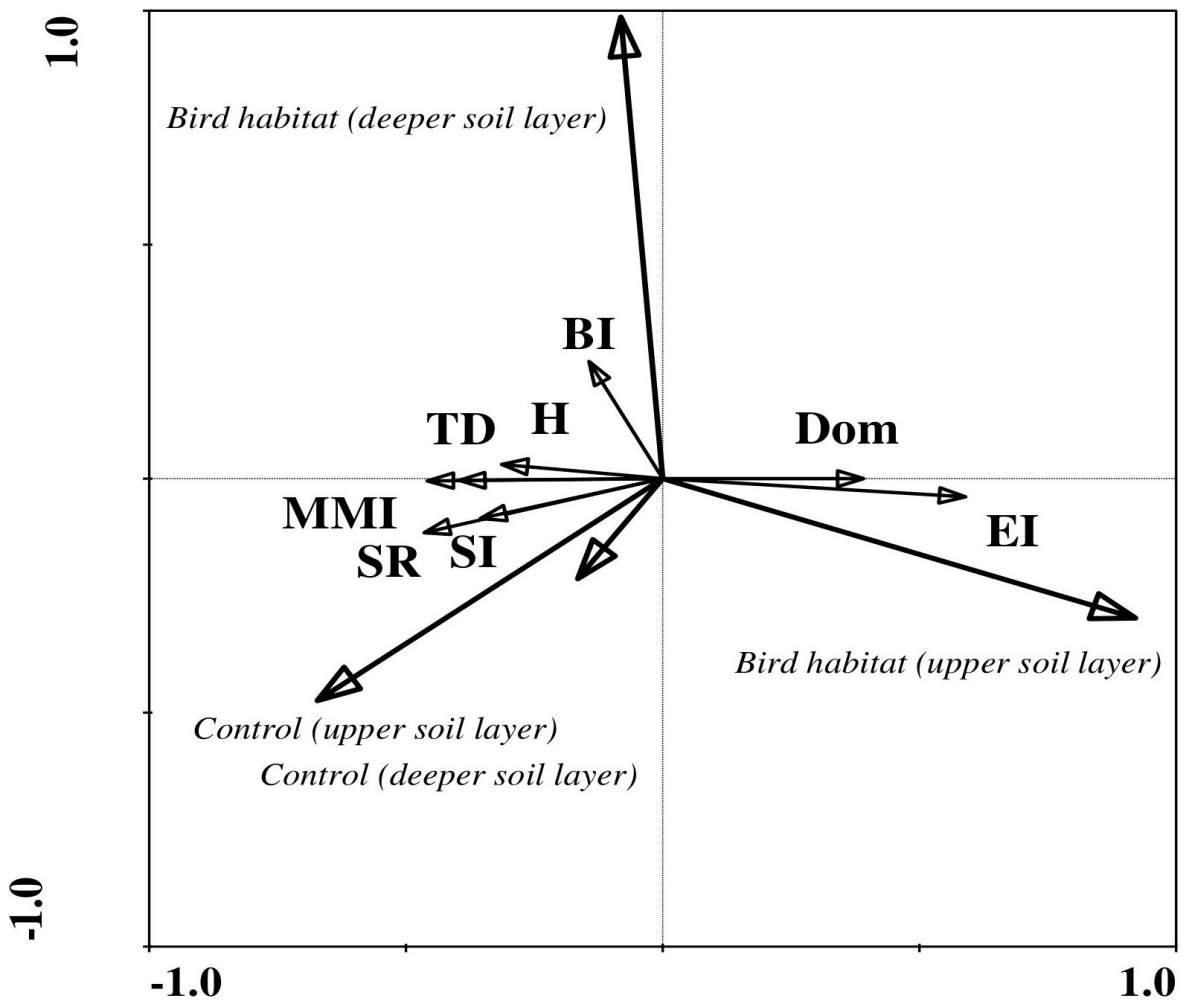
Redundancy analysis (RDA) of ecological index values indicating correlations between habitat variables and soil biotic community structure during the wet period in the study area. Ecological indices showed significant differences between bird habitats and uninhabited control sites in both soil layers. Arrow lengths and angles indicate strength and degree of correlation between the ecological indices and the environment. The first axis of the figure explains 19% of the total variance in the observed data, with the sum of all canonical eigenvalues amounting to 21%. The significance of these variations was confirmed by the Monte Carlo permutation test (*p* = 0.002, *F* = 12.30, where number of permutations = 499). Location: control site, upper soil layer (0–5 cm); control site, deeper soil layer (5–10 cm); bird habitat, upper soil layer (0–5 cm); bird habitat, deeper soil layer (5–10 cm). Ecological indices: TD, trophic diversity; Dom, Simpson’s dominance index; H′, Shannon index; MMI, modified maturity index; SR, species richness; SI, structure index; BI, basal index; EI, enrichment index.

The ecological indices correlated more often with number of microorganisms, NH_4_, and P in the bird-inhabited sites, and with soil moisture, pH, number of bacteria and P in the control sites during the wet period (Table 6). Finally, negative correlations prevailed in the bird-inhabited vs. control sites (Table 6). Statistical analysis showed that the values of some indices (Simpson’s dominance, basal and enrichment) were higher in bird-inhabited plots, in contrast to others (trophic diversity, Shannon, modified maturity, structure) with higher values in control plots during the wet study period (Figure 2).

### 3.5. Seasonal differences between dry and wet (this study) periods

#### 3.5.1. Soil properties

**Figure 3:**
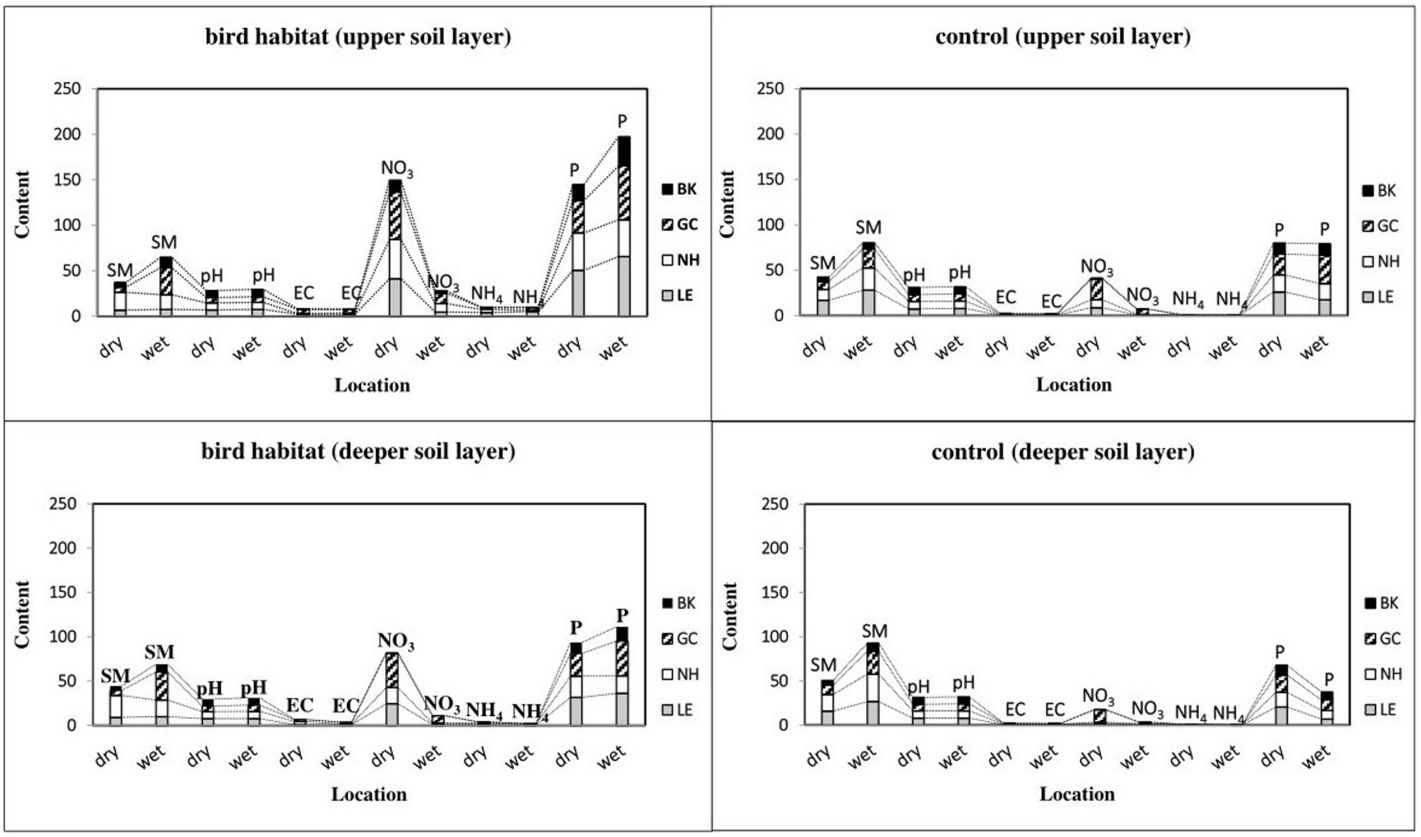
Changes in soil properties in colonial birds’ habitat and control sites during seasonal dry and wet periods. Bird species: LE, little egret (*Egretta garzetta*); NH, black-crowned night heron (*Nycticorax nycticorax*); GC, great cormorant (*Phalacrocorax carbo*); BK, black kite (*Milvus migrans*). Soil properties: SM, soil moisture; pH, soil acidity and alkalinity; EC, electrical conductivity (dS/m); NO_3_, nitrate (mg/100 g dry soil); NH_4_, ammonium cation (mg/100 g dry soil); P, phosphorus (mg/100 g dry soil). One-way ANOVA, and nonparametric Kruskal–Wallis test (*p < 0.05, **p < 0.01, ***p < 0.001, *n* = 128) indicate differences between all compared birds’ habitat and control sites.

Mean soil moisture content was notably higher for great cormorant (in the control and inhabited sites) in the wet vs. dry period in both soil layers (*p* < 0.001 and 0.004, respectively; Figure 3; Pen-Mouratov and Dayan, 2019). In addition, soil moisture content was higher in the control sites for little egret (upper soil layer, *p* < 0.03) and black-crowned night heron (deeper soil layer, *p* < 0.003) in the wet vs. dry period, with no differences between compared sites in the black kite area (Figure 3).

Unlike soil moisture, pH was lower in all of the observed bird-inhabited sites, except in the dry period for black-crowned night heron and black kite (Figure 3). Moreover, soil pH was lower for the little egret-inhabited site in both soil layers (*p* < 0.001 and *p* < 0.03, respectively) and for the great cormorant-inhabited area in both soil layers (*p* < 0.001 and *p* < 0.03, respectively) in the dry vs. wet period, but was higher for the black-crowned night heron habitat (deeper soil layer, *p* < 0.04; Figure 3). Soil pH was also higher in the black kite habitat (both soil layers, *p* < 0.03 and *p* < 0.02, respectively) in the wet vs. dry period (Figure 3). Furthermore, pH was higher in the little egret control site (upper soil layer, *p* < 0.0001) and the great cormorant (upper soil layer, *p* < 0.03; deeper soil layer, *p* < 0.005), but lower for the black kite control site (deeper soil layer, *p* < 0.01) in the wet vs. dry period, with no differences between compared sites in the other bird areas (Figure 3).

Compared to the dry period, the EC values during the wet period were lower in the black-crowned night heron habitat (both soil layers, *p* < 0.0003 and *p* < 0.0001, respectively), black kite habitat (upper soil layer, *p* < 0.03) and little egret habitat (deeper soil layer, *p* < 0.001; Figure 3). EC was higher in the little egret control site (upper soil layer, *p* < 0.04^KW^) in the wet vs. dry period, but lower in the great cormorant control site (upper soil layer, *p* < 0.03; Figure 3).

NO_3_ content was higher in all observed sites (*p* < 0.05), except for the little egret control site (deeper soil layer) and the black-crowned night heron control site (deeper soil layer), in the dry vs. wet period (Figure 3). Similarly, NH_4_ content was higher in the dry period in the deeper soil layer (*p* < 0.05), but in the upper soil layer, it was only higher in the black-crowned night heron habitat (*p* < 0.01), and lower for the other inhabited areas (*p* < 0.01; Figure 3). Compared to the dry period, *p* values during the wet period were higher in the great cormorant-inhabited area in both soil layers (*p* < 0.05) and in the black kite-inhabited area (*p* < 0.0001) in the upper soil layer, and lower (*p* < 0.005) for the black-crowned night heron-inhabited area in the deeper soil layer, with no differences between other compared sites (Figure 3). In contrast to the upper soil layer. P content in the control areas in the deeper soil layer during the dry period were higher (*p* < 0.05) for all observed bird colonies than during the wet period (Figure 3).

#### 3.5.2. Colony-forming units of fungi and bacteria

**Figure 4:**
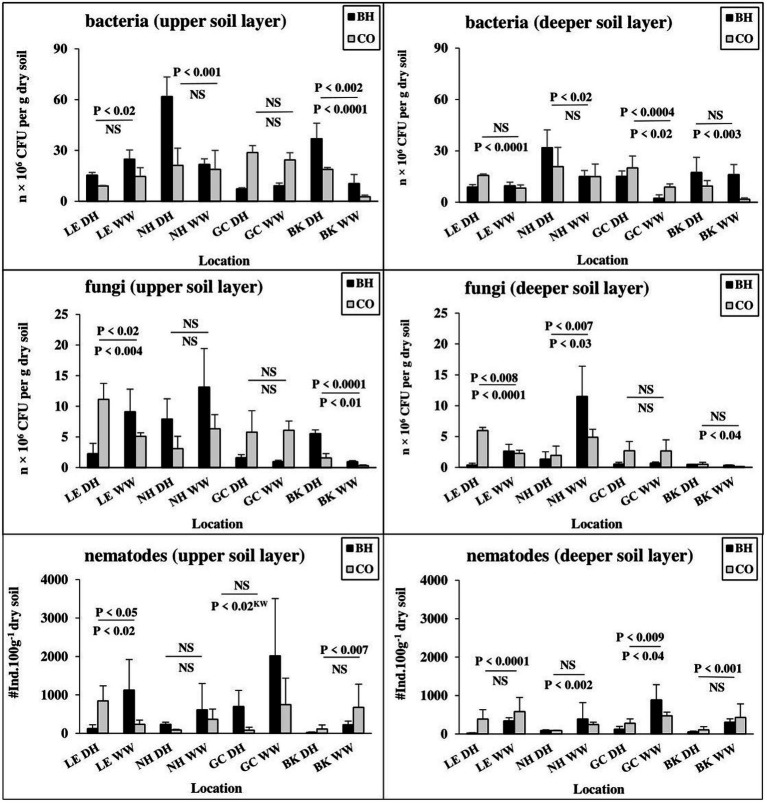
Changes in abundance of soil biota in colonial birds’ habitat (BH) and control (CO) sites during seasonal dry (DH) and wet (WW) periods. Bird species: LE, little egret (*Egretta garzetta*); NH, black-crowned night heron (*Nycticorax nycticorax*); GC, great cormorant (*Phalacrocorax carbo*); BK, black kite (*Milvus migrans*). Abbreviations: u.s.l., upper soil layer; d.s.l., deeper soil layer. Soil biota: TN, total free-living nematodes; colony-forming units (CFU) of bacteria (*B*, *n* × 10^6^ CFU per g dry soil) and fungi (*F*, *n* × 10^6^ CFU per g dry soil). One-way ANOVA and nonparametric Kruskal–Wallis test (*p < 0.05, **p < 0.01, ***p < 0.001, *n* = 128) indicate differences between all compared BH and CO sites. Significance levels divided by a line indicate seasonal differences between BH (upper) and CO (lower) sites.

Bacterial CFU showed seasonal differences between dry and wet periods in the upper soil layer in all observed bird sites (except for the great cormorant habitat). In the deeper soil layer, bacterial numbers differed seasonally only for the black-crowned night heron and great cormorant habitats and for the little egret, great cormorant and black kite control sites (Figure 4). In contrast to the wet period, bacterial CFU in the dry period was lower in the bird-inhabited vs. control sites for the little egret and higher for the black-crowned night heron (both soil layers), black kite (upper soil layer) and great cormorant (deeper soil layer; Figure 4). In contrast, fungal CFU showed seasonal differences between dry and wet periods only for little egret and black kite in the upper soil layer, and little egret, black-crowned night heron and black kite (control sites only) in the deeper soil layer (Figure 4).

The Wardle–Parkinson change index revealed both stimulatory and inhibitory effects of the observed birds on soil microorganisms in the study area during the study periods (Table A1). Among the observed colonial birds, great cormorant activity had the strongest inhibitory effect on soil microorganisms during the observed periods (Table A1). Little egret exerted an inhibitory effect on microorganisms mainly in the dry period, and a stimulatory or neutral effect during the wet period (Table A1). In contrast, black-crowned night heron and black kite colonies had mainly positive effects on soil microorganisms in both periods (Table A1).

#### 3.5.3. Nematode community structure

During the dry period, in contrast to the wet period, the total number of nematodes was significantly lower in the upper soil layer of the little egret-and black kite-inhabited sites compared to the corresponding control sites, whereas in the black-crowned night heron and great cormorant habitats at the same depth, it was higher than in the corresponding control sites (Figure 4). In the deeper soil layer, the total number of nematodes was significantly lower only for little egret-and great cormorant-inhabited vs. the corresponding control sites, and showed no difference between other compared sites (Figure 4). In addition, the total number of nematodes showed seasonal differences between dry and wet periods for little egret areas (both control and bird-inhabited), great cormorant control sites and black kite-inhabited sites) in the upper soil layer, whereas in the deeper soil layer, a seasonal difference was found for little egret (inhabited site), black-crowned night heron (control site), great cormorant and black kite (inhabited sites), with a tendency toward higher total number of nematodes during the wet period (Figure 4).

**Figure 5:**
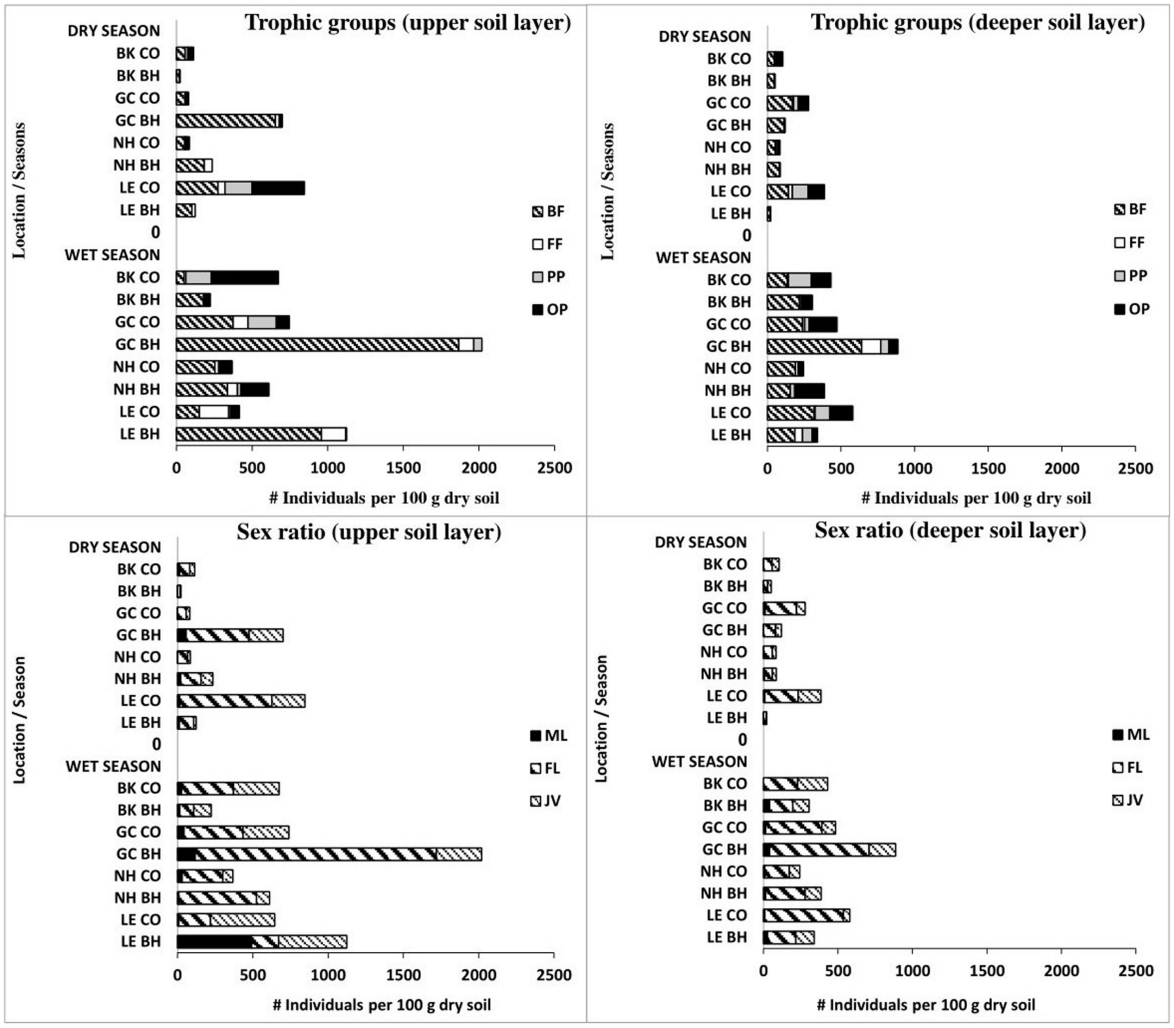
Seasonal changes in trophic groups and sex ratio of soil free-living nematodes in colonial birds’ habitat (BH) and control (CO) sites during seasonal wet and dry periods. Bird species: LE, little egret (*Egretta garzetta*); NH, black-crowned night heron (*Nycticorax nycticorax*); GC, great cormorant (*Phalacrocorax carbo*); BK, black kite (*Milvus migrans*). Trophic groups of nematodes: BF, bacteria-feeding; FF, fungi-feeding; PP, plant-parasitic; OP, omnivore-predator. Sex groups of nematodes: ML, male; FL, female; JV, juvenile.

Overall for the two studied periods (wet here and dry in Pen-Mouratov and Dayan, 2019), we identified 81 nematode taxa: 29 (26 and 21 in the dry and wet periods, respectively) belonged to the bacteria-feeding trophic group, 7 (6 and 7 in the dry and wet periods, respectively) were fungi-feeding nematodes, 22 (17 and 15 in the dry and wet periods, respectively) were plant-parasitic nematodes, and 23 (13 and 18 in the dry and wet periods, respectively) were omnivore-predator nematodes (Table A2). Along with the total number of nematodes, bird activity was also found to affect the trophic and sexual structures of the nematode community during both wet and dry periods (Figure 5).

Of the 81 nematode taxa identified in this study, 20 were found only in the dry season, and another 18 only in the wet season, while the remaining nematodes occurred in both the dry and wet periods of the year (Table A2). In contrast to the dry period, the nematode sex ratio did not undergo any significant changes (except for males in the upper soil layer of the little egret area) under the influence of bird activity in the wet period (Figure 5). The sex ratio revealed different correlations with the observed soil properties and soil microorganisms during the two observed seasons.

**Figure 6:**
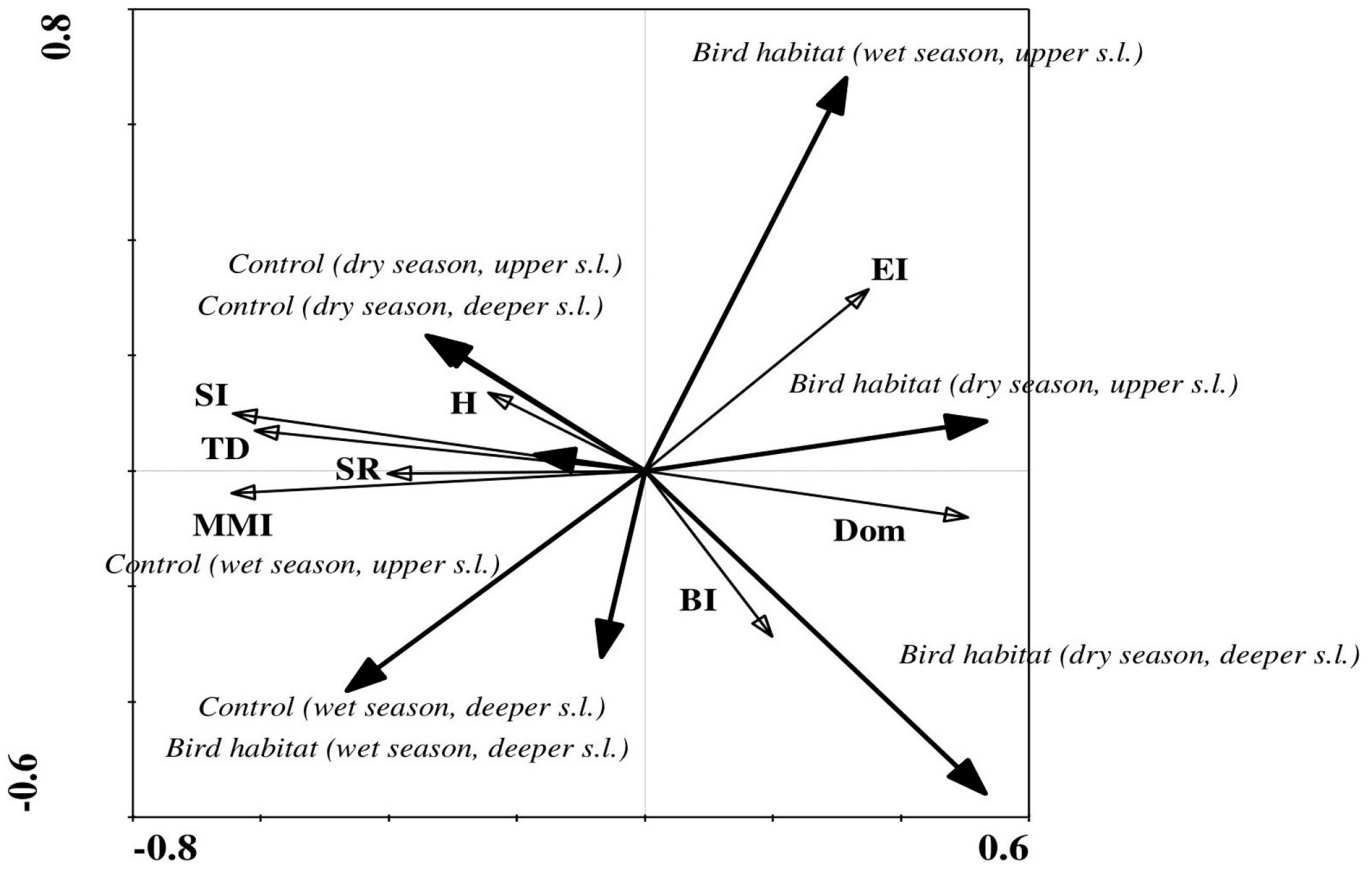
Redundancy analysis (RDA) of ecological index values indicating correlations between habitat variables and soil biotic community structure during the seasonal dry and wet periods in the study area. Ecological indices showed significant differences between bird habitats and uninhabited control sites in both soil layers. Arrow lengths and angles indicate strength and degree of correlation between the ecological indices and the environment. The first axis of the figure in panels A and B explains 36% and 19% of the total variance in the observed data, with the sum of all canonical eigenvalues amounting to 37% and 21% in the dry and wet periods, respectively. The significance of these variations was confirmed by Monte Carlo permutation test (*p* = 0.002, *F* = 11.95 for the dry period and *p* = 0.002, *F* = 12.30 for the wet period, where number of permutations = 499). Location: control site, upper soil layer (0–5 cm); control site, deeper soil layer (5–10 cm); bird habitat, upper soil layer (0–5 cm); bird habitat, deeper soil layer (5–10 cm). Ecological indices: TD, trophic diversity; Dom, Simpson’s dominance index; H′, Shannon–Weaver index; MMI, modified maturity index; SR, species richness; SI, structure index; BI, basal index; EI, enrichment index.

RDA of the ecological index values showed significant differences between bird-inhabited and control sites, as well as between the observed seasonal dry and wet periods (Figure 6). In contrast to the nematode faunal indices—Simpson dominance, basal and enrichment—that showed higher values in the bird habitats, the other generally accepted ecological indices — Shannon–Weaver, modified maturity, species richness and structure—had higher values in the control sites during the dry season in both upper and deeper soil layers (Figure 6). However, in the wet season, only the enrichment and dominance index values (in the upper soil layer) were higher in the bird-inhabited areas; the other applied ecological indices had higher values in the control sites (Figure 6). At the same time, in contrast to the dry period, the basal index, which is used as an indicator of a food web diminished by stress or by limited nutrient resources, showed higher values in the control sites during the wet period in the deeper soil layer (Figures 2, 6).

## 4. Discussion

Animal activities (Steinberger et al., 2004), and in particular those of colonial birds (Pen-Mouratov and Dayan, 2019; Pen-Mouratov, 2022), can affect soil ecosystems and their inhabitants through the input of allochthonous matter, resulting in the formation of spatiotemporal heterogeneity of soil properties in the study area (Pen-Mouratov and Dayan, 2019). However, seasonal fluctuations can affect and even weaken the impact of animals’ activity on the structure, abundance and diversity of soil biota in particular, and the soil ecosystem in general (Pen-Mouratov et al., 2019). The present study, in accordance with previous results, confirmed the effect of seasonal fluctuations on the impact of birds’ excrement on the soil biota during the observed periods (Ilieva-Makulec et al., 2015; Pen-Mouratov and Dayan, 2019; Pen-Mouratov, 2022). In contrast to findings obtained in the dry period, during the wet period, most of the surveyed bird species’ activities stimulated the abundance of soil microorganisms, except for the great cormorant which had an inhibitory effect (Pen-Mouratov, 2022).

In contrast to the more acidic medium in the bird habitat during the dry period (Pen-Mouratov and Dayan, 2019), strong differences in pH values were found between all compared bird sites and control areas in the wet period. The black kite area had the most acidic medium among the observed bird-inhabited sites. In addition, changes in the alkaline–acid balance of the soil and its subsequent impact on the number of soil microorganisms were correlated with seasonal fluctuations: soil microorganism level was strongly negatively correlated to pH in the little egret and black-crowned night heron (bacterial communities) and little egret and great cormorant (fungal communities) habitats during the dry period (Pen-Mouratov and Dayan, 2019), whereas this same influence was seen in the little egret and great cormorant (bacterial communities) and great cormorant (fungal communities) habitats during the wet period. Previous research has shown that microorganisms in fine-textured soils are more protected from predation than those in coarse-textured soils due to pore size restricting predator activity, as well as to differences in nutrient content (Hassink et al., 1993; Pen-Mouratov et al., 2011). However, the differences in nutrient content between the piscivorous and omnivorous birds’ colonies varied seasonally, decreasing toward the wet period. Hence, the protective ability of the fine-textured soils (piscivorous birds’ habitat) compared to the coarse-textured soils (omnivorous birds’ habitat) strongly depends on seasonal changes and can be attenuated by seasonal fluctuations. Furthermore, our previous study, conducted during the dry period (Pen-Mouratov and Dayan, 2019), indicated that the relationships between soil microorganisms and soil nematodes are strongly dependent on soil conductivity; however, this dependence was not observed during the wet period. We therefore conclude that soil conductivity, along with soil pH, is one of the main drivers of prey–predator relationships in the observed birds’ habitats during both dry and wet periods, despite a marked attenuation in its effect during the latter period.

In accordance with earlier findings (e.g., Otero et al., 2018; Bogatyreva et al., 2022), the contents of NO_3_, NH_4_ and P were high in the bird excrement (Pen-Mouratov and Dayan, 2019). In some cases, the differences in chemical contents between bird and control sites reached significantly high values. Input of NO_3_ into the ecosystem by all of the observed colonies was higher in the dry period than in the wet one (Pen-Mouratov and Dayan, 2019), whereas the input of NH_4_ and P was dependent on the bird species diversity and varied seasonally between different bird sites in the two soil layers. It is obvious that changes in the soil chemical content of bird sites are strongly dependent on the activity and diversity of the bird communities and subject to seasonal fluctuations. In turn, changes in soil properties caused by the birds’ activity, associated with seasonal fluctuations, were reflected in the abundance of soil microorganisms and in the abundance and diversity of soil nematodes, revealing a tight correlation of the soil biota with the observed soil properties and seasonal fluctuations during the study periods. In addition, different bird species during the dry and wet periods had variable impacts on the habitat, abundance, trophic composition and genera of soil nematodes. During the hottest period of the year, when external adverse factors dominate the birds’ activity, a stronger impact was exerted on the nematode communities. Our previous results revealed a negative effect of little egret, black kite and great cormorant (upper soil layer) and a positive effect of black-crowned night heron (upper soil layer) and great cormorant (deeper soil layer) on free-living nematode abundance and diversity during the dry period (Pen-Mouratov and Dayan, 2019). In the wet period, when seasonal fluctuations decrease the impact of the birds’ activity, a positive effect on the abundance of nematode communities was only found in the little egret and great cormorant habitats. Furthermore, during the wet period, there was a tendency toward increasing abundance of soil nematodes. Our results suggest that the birds’ activity exerts significant effects (through changes in soil properties) on soil free-living nematodes. In addition, during the dry period, the chemical effect on nematode abundance was notably stronger than during the wet period.

Numerous studies have shown that soil ecosystem disturbances, along with nematode density, have a significant impact on the species and trophic diversity of the nematode communities (e.g., Bongers and Bongers, 1998). Moreover, seasonal fluctuations have a variable impact on these communities, with significant attenuation of the effect of animal activity on their abundance and diversity (Pen-Mouratov et al., 2019). In agreement with previous research, our results obtained over two seasons indicate that abundance of the observed nematodes increases markedly in the wet vs. dry period. Moreover, more than 50% of the observed free-living soil nematodes found the bird sites less suitable during both wet and dry seasons. Among them, the bacteria-feeding and fungi-feeding nematodes were less sensitive to bird activity than the plant-parasitic and omnivore-predator nematodes, consistent with previous findings (e.g., Ilieva-Makulec et al., 2018; Pen-Mouratov and Dayan, 2019). In general, nematodes belonging to the *r*-strategist group (colonizers, tolerant to environmental disturbance), mainly bacteria-feeding (BF1 and BF2), were the most numerous in the study area, varying from 89 to 77% in the bird sites during the dry and wet periods, respectively, and from 44 to 46% in the control sites during these respective periods. The plant-parasitic and omnivore-predator nematodes were the smallest groups, numerically, in the bird areas during the dry period, with a gradual increase during the wet period. Unlike the other trophic groups, the fungi-feeding nematodes did not show any significant differences in numbers between the bird areas and their corresponding control sites, or between the compared seasons. These results are consistent with previous research showing that different animals exert a significantly different seasonal effect on the abundance and diversity of nematodes belonging to different functional guilds (Pen-Mouratov et al., 2019). The nematodes belonging to the *r*-strategist group were dominant in all bird areas. The omnivore-predator nematodes belonging to the *K*-strategist group (e.g., Bongers and Bongers, 1998), were rare in the bird areas: only *Microdorylaimus* was found in the bird sites during the dry period (Pen-Mouratov and Dayan, 2019). However, the attenuating effect of seasonal fluctuations in the wet period led to the appearance of nine more *K*-strategists in the bird-inhabited areas, with *Microdorylaimus* and *Dorylaimoides* dominating the omnivore-predator nematode community. In addition to previous findings, it should be noted that the close relationship between the observed sex groups of nematodes and soil microorganisms was only characteristic of the dry period (Pen-Mouratov and Dayan, 2019). During the wet period, these relationships weakened significantly, and confirmation of soil fungi as an important food source for female nematodes was only obtained in the black-crowned night heron habitat during this period. Moreover, soil fungi were found to be a significant food source for male nematodes in the great cormorant habitat, along with soil bacteria for female and juvenile nematodes in the black kite control site during the wet period. The effect of soil properties, including soil acidification, on the sex ratio of soil free-living nematodes was also significantly reduced, and was not detected at all in the bird-inhabited sites during the wet period.

The ecological indices applied in the current study confirmed that the different species of colonial birds’ droppings exert different (stimulatory or inhibitory) impacts (mainly through changes in soil properties) on the abundance and diversity of the soil biota, affecting the structure of soil free-living nematodes at the generic, trophic and sexual levels. In addition, the seasonal wet–dry fluctuations may significantly change the extent of the impact of colonial birds’ droppings. In the present study, the trophic diversity index and species richness values confirmed that the colonial birds’ sites were unfavorable habitats for soil biota compared to the control sites. However, the birds’ impact diminished with depth during the wet period. For example, the diversity indices demonstrated a decrease in the contribution of rare species and an increase in the contribution of common species during the dry period (Pen-Mouratov and Dayan, 2019). However, in the wet period, the indices demonstrated a decrease in the contribution of rare species only in the upper soil layer for little egret and black kite habitats, and an increase in the contribution of common species only in the upper soil layer for the black kite habitat.

Maturity indices indicated that the bird-inhabited sites are less suitable for soil biota compared to the corresponding control sites. They further revealed that the negative impact of the birds’ activity on soil nematode communities can be weakened by seasonal fluctuations. The Wardle–Parkinson change index (Neto et al., 2012) showed that different bird species’ droppings exert significantly different effects on soil nematode density, with a more negative impact during the dry period and a weakening of these effects during the wet period due to seasonal fluctuations. The food-web indices—basal, structure and enrichment—showed that the bird sites are characterized by a less complex food web and more plentiful nutrient resources than the uninhabited control areas, but with a strong dependence on seasonal fluctuations. The structure index (Ferris et al., 2004) suggested the presence of a food web with more trophic linkages, but showed a decline in trophic linkages in bird-inhabited areas. However, the structure index indicated that the negative impact on the food web can be weakened by seasonal fluctuations. The basal index, which is used as an indicator of food webs (Ferris et al., 2001), demonstrated increasing resource availability only in the little egret and black kite areas during the dry period and in the little egret habitat and control site for the great cormorant during the wet period. Moreover, the basal index showed that the different soil properties exert different seasonal effects on the diversity of the trophic linkages in the study area. The enrichment index (Ferris et al., 2004) indicated that the interaction between primary decomposers and soil resources is more effective in the bird-inhabited areas during the wet period, and that during the dry period, it is mainly negatively dependent on pH in all habitats (except the little egret habitat). In contrast, there was a positive correlation with the soil nutrients in all observed bird sites except for the black-crowned night heron habitat during the wet period.

## 5. Conclusion

Droppings of the different species of colonial birds were found to exert different (stimulatory or inhibitory) effects (mainly through changes in soil properties) on the number of soil microorganisms (bacterial and fungal communities) and the abundance and diversity of the soil free-living nematodes, affecting the structure of soil nematodes at the generic, trophic and sexual levels. In addition, the seasonal wet–dry fluctuations may significantly change the extent of the colonial birds’ impact. The ecological indices confirmed the sensitivity of the free-living nematodes to environmental disturbances caused by the different colonial birds’ activities. Our findings demonstrate that the impact of the birds’ droppings on free-living nematode communities should be examined in different seasons to take into account the attenuating effects of seasonal fluctuations.

## Data availability statement

The raw data supporting the conclusions of this article will be made available by the authors, without undue reservation.

## Author contributions

All authors listed have made a substantial, direct, and intellectual contribution to the work and approved it for publication.

## Funding

This study was funded by KAKMAB. KAKMAB is a support system covering additional payments for the research activity of the researchers in Tel Aviv University.

## Conflict of interest

The authors declare that the research was conducted in the absence of any commercial or financial relationships that could be construed as a potential conflict of interest.

## Publisher’s note

All claims expressed in this article are solely those of the authors and do not necessarily represent those of their affiliated organizations, or those of the publisher, the editors and the reviewers. Any product that may be evaluated in this article, or claim that may be made by its manufacturer, is not guaranteed or endorsed by the publisher.
